# Testing health information technology tools to facilitate health insurance support: a protocol for an effectiveness-implementation hybrid randomized trial

**DOI:** 10.1186/s13012-015-0311-4

**Published:** 2015-08-25

**Authors:** Jennifer E. DeVoe, Nathalie Huguet, Sonja Likumahuwa-Ackman, Heather Angier, Christine Nelson, Miguel Marino, Deborah Cohen, Aleksandra Sumic, Megan Hoopes, Rose L. Harding, Marla Dearing, Rachel Gold

**Affiliations:** Department of Family Medicine, Oregon Health & Science University, 3181 S.W. Sam Jackson Park Rd., Portland, OR 97239 USA; OCHIN, Inc., 1881 SW Naito Parkway, Portland, OR 97201 USA; Center for Health Research Northwest, Kaiser Permanente, 3800 N. Interstate Avenue, Portland, OR 97227 USA

**Keywords:** Cancer screening, Health insurance, Medicaid, Health information technology, Primary care, Hybrid design

## Abstract

**Background:**

Patients with gaps in health insurance coverage often defer or forgo cancer prevention services. These delays in cancer detection and diagnoses lead to higher rates of morbidity and mortality and increased costs. Recent advances in health information technology (HIT) create new opportunities to enhance insurance support services that reduce coverage gaps through automated processes applied in healthcare settings. This study will assess the implementation of insurance support HIT tools and their effectiveness at improving patients’ insurance coverage continuity and cancer screening rates.

**Methods/design:**

This study uses a hybrid cluster-randomized design—a combined effectiveness and implementation trial—in community health centers (CHCs) in the USA. Eligible CHC clinic sites will be randomly assigned to one of two groups in the trial’s implementation component: tools + basic training (Arm I) and tools + enhanced training + facilitation (Arm II). A propensity score-matched control group of clinics will be selected to assess the tools’ effectiveness. Quantitative analyses of the tools’ impact will use electronic health record and Medicaid data to assess effectiveness. Qualitative data will be collected to evaluate the implementation process, understand how the HIT tools are being used, and identify facilitators and barriers to their implementation and use.

**Discussion:**

This study will test the effectiveness of HIT tools to enhance insurance support in CHCs and will compare strategies for facilitating their implementation in “real-world” practice settings. Findings will inform further development and, if indicated, more widespread implementation of insurance support HIT tools.

**Trial registration:**

Clinical trial NTC02355262

## Background

Cancer morbidity and mortality can be greatly reduced through screening and prevention; however, not all patients have access to regular cancer prevention services. In the USA, uninsured populations are much less likely to receive these evidence-based services as recommended, compared to those with insurance coverage [1–8]. Further, when health insurance coverage gaps occur, patients often delay or forgo cancer prevention services [9–13]. These delays in cancer detection and diagnoses lead to higher rates of disease incidence and mortality and increased healthcare costs [2, 14–22]. Patients who regain health insurance coverage are often able to catch up on missed prevention services [9–13]. Thus, interventions that optimize and stabilize health insurance coverage could substantially improve rates of receipt of timely cancer preventive care.

Community health centers (CHCs) are well-positioned to provide such health insurance support because many of their patients are uninsured or experience frequent coverage gaps [23, 24]. Recent advances in health information technology (HIT) create new opportunities for enhancing insurance support services in CHCs and other healthcare settings through automated processes. Since the passage of the Affordable Care Act (ACA) in 2010, there are new insurance programs (i.e., Medicaid expansions) available for socioeconomically vulnerable patients [25]. This confluence of factors presents a window of opportunity to develop, test, and implement health insurance support technologies.

### Health insurance support HIT tools

Most healthcare institutions collect information about patients’ insurance coverage. These data are documented in the electronic health record (EHR) but then are often used exclusively for billing purposes. While many CHCs already engage in health insurance support, few have an automated way to use patients’ health insurance data to identify, track, or communicate with patients about their health insurance status. We built insurance support HIT tools that incorporated information already in the EHR, augmented by the collection of a few additional data elements. These insurance support HIT tools were modeled on those proven effective for chronic disease management such as a computer reminder system for colorectal cancer screening [26–28]. The tools include a panel management/data aggregator system, which identifies patients who may be eligible for Medicaid coverage but are not yet insured or who may be nearing coverage expiration.

In recent pilot studies, we showed that these insurance support HIT tools can be integrated into clinic workflows in several ways [29–31]. The tools can provide “pop-up” alerts that appear at check-in or while scheduling an appointment. The tools also create registry lists of patients who are uninsured or may be nearing insurance expiration dates. These registries provide clinic staff with information about patients to contact and offer insurance enrollment support. Registries also can be used to support automatic messages (e.g., e-mail, voicemail, texts, after-visit communications) to remind patients about upcoming insurance re-enrollment dates and provide them with resources.

### Effectiveness-implementation hybrid study design

To further study the uptake and use of these health insurance support technologies, we will blend design components of clinical effectiveness and implementation research. Using an “effectiveness-implementation hybrid design,” as described by Curran and collaborators [32], this intervention study will simultaneously assess the effectiveness of (i) health insurance support HIT tools and (ii) the best strategies for implementing the tools in CHCs. We hypothesize that patients seen at CHCs that receive these insurance support HIT tools will have higher rates of continuous insurance coverage and will be more up-to-date on age- and gender-appropriate cancer screening and prevention services, as compared to those seen at CHCs without the tools. We further hypothesize that CHCs that receive enhanced training and facilitation to support tool use will have higher rates of uptake of the tools and better insurance and cancer prevention rates than CHCs that receive the tools without such enhanced training or those without the tools.

Specifically, we will assess the effectiveness and implementation of a suite of health insurance support HIT tools designed to (1) identify and assist in contacting uninsured CHC patients who are eligible for enrollment in Medicaid and (2) encourage re-enrollment of Medicaid-insured patients before coverage gaps occur. The current study builds on our preliminary work by refining and studying the effectiveness of EHR-based health insurance support HIT tools across a larger number of clinics and studying effective methods for implementation of such tools in an adult patient population [30, 33–35].

### Study objectives

1. Assess the effect of the health insurance support HIT tools on patients’ health insurance coverage rates.
2. Assess the effect of the health insurance support HIT tools on up-to-date status of cancer screening and preventive care received by patients.
3. Compare two levels of implementation support, evaluating patient and CHC staff acceptance and use of the health insurance support HIT tools, and patient-, provider-, and system-level factors associated with successful implementation of the tools.

## Methods/design

### Trial design

**Fig. 1:**
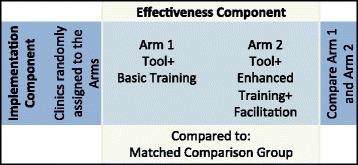
Effectiveness-implementation hybrid design

This is a two-arm, cluster-randomized trial with an external matched control group of clinics, which utilizes a hybrid design: an effectiveness and implementation trial [32, 36–38]. To test for *implementing the tools*, intervention clinics will be randomized to one of the two implementation support arms (see Fig. 1). Thus, all intervention clinics will receive the health insurance support HIT tools but will be randomly assigned to Arm I (tool + basic training) or Arm II (tools + enhanced training + facilitation) as depicted in Fig. 1. The *implementation* component of the trial will evaluate the relative impact of the different support strategies in Arm II. For the *effectiveness* component of the trial, we will compare the intervention group (all participating clinics) to a propensity score-matched [39–41] group of control clinics to assess the extent to which the use of insurance support HIT tools facilitates continuous health insurance coverage and improved cancer prevention care.

### Study setting and population

The study will be carried out at OCHIN, a non-profit 501(c)(3) collaborative that was created in 2001 to develop HIT tools for CHCs. Originally called the Oregon Community Health Information Network, OCHIN has 81 health system members in 17 states with 376 clinics and 6322 providers caring for nearly 2,000,000 patients.

OCHIN provides and maintains a comprehensive electronic health information infrastructure, built on software from Epic© Systems. The OCHIN EHR is linked across all member clinics through an Organized Health Care Arrangement, which gives clinics a fully integrated EHR in which each patient has a single medical record. There is one enterprise-wide master patient index; health record data “follow the patient” to any OCHIN clinic.

The Institutional Review Board of the Oregon Health & Science University has reviewed and approved this study. The study is registered with ClinicalTrials.gov (#NCT02355262).

*Inclusion criteria for intervention and control sites*. Study sites were required to be primary care clinics that implemented the OCHIN EHR prior to January 1, 2013, have >1000 adult patients with ≥1 visits in the past year, and be located in a state that expanded Medicaid in 2014 [42].

*Inclusion criteria for patients*. All patients (ages 18–64) with at least one encounter at a CHC in OCHIN since January 1, 2013 (*n* ≈ 1,000,000 adults), will be included in the study dataset for quantitative analysis. As very different insurance products are available to children <18 and patients >64 years of age, these age groups are excluded. All study sites serve low-income, ethnically diverse populations with lower rates of cancer screening (see Table 1) and higher rates of publicly insured and uninsured patients, compared to national rates [43].

**Table 1:** Insurance coverage and cancer screening rates in the US population and OCHIN

	US population, 2010 (%)	OCHIN, 2014 (%)
Insurance coverage		
Private	64.2	17.6
Public	34.3	48.4
None	13.4	34.0
Cancer screening among insured patients		
Cervical	83.0	36.5
Colorectal	58.6	42.8
Breast	72.4	60.3
Cancer screening among uninsured patients		
Cervical	63.8	27.0
Colorectal	20.7	19.9
Breast	38.2	39.3

Source for US population: Census [71], CDC [72]; source for OCHIN population: OCHIN EHR data

### Overview of implementation component of trial

#### Intervention sites

An OCHIN team member identified 99 clinics in 32 eligible health center organizations within OCHIN. Of those invited, 23 clinics in 7 health center organizations agreed to participate (23 % of eligible clinics) and 76 clinics in 25 organizations declined (77 % of eligible clinics). The 7 participating organizations are located in 3 Medicaid expansion states (Oregon, California, and Ohio). These organizations varied in size; the number of primary care clinic sites per organization ranged from 1 to 6. Due to the possible correlation among clinic sites in the same organization, an independent biostatistician randomized the 23 clinics by service organization. A covariate-constrained cluster randomization [44] was used to balance the number of clinics between the two treatment arms. The randomization was balanced using the following covariates: state the organization was located in (Oregon vs. non-Oregon), total number of patients per organization, and percentage of uninsured patients per organization. The biostatistician and research team members were blind to the clinics during randomization. Once randomized, the study team was given the list of clinics participating in each study arm (11 in Arm I and 12 in Arm II).

#### Pre-implementation phase

We will first assess baseline rates of insurance coverage, cancer screening, and general recommended preventive care across all study arms and the matched controls. We will also assess rates of these outcomes across the total OCHIN population to compare to the recruited clinics. Additional analyses including patients from all CHCs in OCHIN will be conducted to assess the impact of recent policy changes on insurance coverage and cancer preventive care. Data for these analyses will be extracted from the OCHIN EHR and will include demographic information (i.e., age, federal poverty level, race/ethnicity, language spoken), insurance coverage dates and types, as well as cancer screenings (e.g., fecal occult blood test (FOBT) orders, colonoscopy and mammography referrals, and pap smear receipt), immunizations (e.g., human papillomavirus (HPV) vaccination), cancer-related health behavior assessment and counseling (e.g., smoking screening and intervention and obesity screening and intervention), and receipt of general recommended preventive care (e.g., blood pressure, glucose, and lipid assessments). This information will be extracted using standardized codes for diagnoses, labs, medications, procedures, and referrals, and electronically collected vital signs and social history (e.g., smoking status) from visits. As seen in Table 2, we will define receipt of cancer screenings and prevention based on services with an A–B rating from the US Preventive Services Task Force and recommendation from the Advisory Committee on Immunization Practices [45, 46]. Our EHR dataset will also contain additional information about the use of health services such as encounter information, provider data, clinic location, reason for visit, level of service, and type and number of visits.

**Table 2:** Description of outcome measures

Dependent variable	Description/measure for preventive care
Insurance coverage	Covered at visit (yes, no); all visits covered vs. not covered; percent of visits covered
Insurance continuity	Months covered by Medicaid; percentage of time covered by Medicaid
Recommended preventive care services (NQF)	Breast cancer screening^a^ (0031), NCQA; currently used by MU1, HEDIS®, and MACS
	Cervical cancer screening^a^ (0032), NCQA; currently used by MU1, HEDIS®, and MACS
	Colorectal cancer screening^a^ (0034); MU1, HEDIS®
	Tobacco use screen and medical assistance with tobacco cessation^a^ (0027), NCQA; currently used by MU1, HEDIS®, and MACS
	Obesity screening and counseling^a^

*NQF* National Quality Forum (www.qualityforum.org), *MACS* Medicaid Adult Core Set (www.medicaid.gov/Medicaid-CHIP-Program-Information/By-Topics/Quality-of-Care/Downloads/Medicaid-Adult-Core-Set-Manual.pdf), *NCQA* National Committee for Quality Assurance (www.ncqa.org), *MU1* Meaningful Use Stage 1 of the Medicare & Medicaid Electronic Health Record Incentive Program (www.cms.gov/Regulations-and-Guidance/Legislation/EHRIncentivePrograms/CQM_Through_2013.html), *HEDIS®* Health Employer Data and Information Set (www.ncqa.org/HEDISQualityMeasurement.aspx), *USPSTF* www.uspreventiveservicestaskforce.org/Page/Name/uspstf-a-and-b-recommendations/

^a^USPSTF A or B recommendation

Additionally, we will conduct an informational phone call with relevant clinic staff (e.g., clinic manager, insurance eligibility specialists) at all intervention sites to learn about the clinic workflows for providing insurance assistance and any other factors that may affect how a clinic implements and uses the insurance support HIT tools. We will share findings from the pre-implementation calls with the HIT tool development team to inform tool development and refinements. These calls will also inform the development of trainings and practice facilitation for Arm II sites. In addition, we will hold a retreat with CHC staff, patient advisors, project advisors and consultants, and policymakers to aid in refining the health insurance support HIT tools. At the retreat, we will utilize user-centered design processes to better understand users’ needs and wants with regard to product design and utilization of the HIT tools [47]. These processes will enable us to interactively share the first version of the tools and develop a work plan and timeline for further refinements. A health literacy expert will assess the reading demands and document complexity of all patient communication materials (e.g., automated e-mails) to ensure that content is appropriate for the CHC patient population.

#### Implementation phase

The HIT tools will be implemented in both study arms, but the arms will differ in the levels of training, facilitation, and implementation support received, as described below.

##### Arm I

Study training materials (e.g., a PowerPoint presentation covering tools and study purpose, a tool guide, sample best practice workflow) will be made available to the clinics in Arm I for independent training via the OCHIN learning management system (LMS). LMS is a software application for the management of documentation, tracking, reporting, and delivery of electronic educational technology, education courses, and training programs.

##### Arm II

In addition to the study training materials provided for Arm I, we will provide interactive trainings for Arm II clinics to explain the insurance support HIT tools, prepare clinic staff for using the tools, and assist clinics in revising workflows to maximize tool utilization. A practice facilitator will be available to the clinic staff and will continue to engage actively with tool users over the course of the implementation phase, including on-site, face-to-face trainings and support.

We will assess the use of the insurance support HIT tools as well as the facilitation process in Arm II qualitatively and quantitatively. We will conduct brief, semi-structured phone interviews with key informants from each Arm II clinic and the facilitator periodically to monitor tasks and workflows related to health insurance support and other factors that may affect how a clinic implements the HIT tools. The goal is to understand what is and is not working regarding the HIT tools, any changes in workflow that might impact insurance support tool use, and experiences with the practice facilitation process. We anticipate conducting 5–10 interviews with each clinic in Arm II. Findings may be used to guide and modify the facilitation process in Arm II and possibly future refinements to the insurance support tools.

### Overview of effectiveness component of the trial

An independent biostatistician used propensity score matching [39] to identify the control group of 23 CHCs that most closely match the intervention clinics on clinic and patient characteristics that have the potential to confound the insurance support HIT tools’ effect on health insurance coverage rates and cancer screening. An OCHIN team member identified 64 clinics in three states that were eligible for the study’s control group and matched based on the state of the organization, the total number of patients in 2014, and the percentage of uninsured visits, female gender, and Medicaid beneficiaries. The 23 control clinics were selected in a 1:1 ratio based on the nearest available match from the intervention clinics. The intent was to achieve the optimal overall balance in the matching characteristics between the intervention and control groups. Balance diagnostics were performed to assess whether the propensity score model had been properly specified [48].

### Post-implementation assessment

After implementation of the insurance support HIT tools, we will assess the effectiveness of these tools and the implementation support strategies using qualitative and quantitative methods.

#### Post-implementation quantitative assessment

We will compare post-implementation insurance coverage and cancer screening and prevention rates across all study arms and in the matched control groups. We will also assess insurance coverage and receipt of general recommended preventive care/cancer screening and prevention across the entire OCHIN population. Study outcome variables are described below (also see Table 2).

#### Post-implementation qualitative assessment

Based on data collected during the implementation period, we will purposively sample CHC sites to participate in site visits. At the site visits, we will evaluate the implementation process, how the HIT tools are being used by clinics and how workflows around insurance are changing, and facilitators and barriers to implementing and using the HIT tools. Based on the quantitative data (described above), we will explore differences between high and low performers across arms.

During each site visit, the research team will spend 3–5 half-days observing clinic operations that are pertinent to use of the insurance support HIT tools. This will include observing all operational areas (e.g., scheduling/check-in, provider encounters) where insurance issues arise and are addressed. The team will also opportunistically conduct informal interviews with clinic staff, as informal interviews often elicit different and more insightful information than is captured during formal interviews. Between 5 and 10 interviews will be conducted at each site. Researchers will prepare field notes, share observations, and strategize for additional data collection opportunities.

If quantitative analyses reveal that control clinics have significantly improved rates of coverage, similar to the intervention clinics, we will employ ethnographic methods to better understand what is contributing to their improvements.

### Study measures

#### Study measures for the effectiveness component of the trial

Our *outcome measures* (presented in Table 2) are patients’ health insurance status and continuity rates and receipt of recommended cancer screenings and prevention (i.e., cervical, colorectal, and breast cancer screening, HPV vaccination, and smoking and obesity screening and intervention). Outcomes will be measured individually and in combination. For example, we will measure if cancer screening services were received within appropriate intervals and also the overall rate of indicated services received.

The primary *independent variable* is provision of insurance support HIT tools (i.e., intervention clinics with tools, control clinics without tools). Potential *confounder variables* in multivariable analyses will include patients’ demographic information (e.g., age, federal poverty level, race/ethnicity, language spoken, rural/urban location). We will also collect additional information from the EHR about the use of health services such as encounter information, provider data, clinic location, level of service, types of visits, and number of visits.

#### Study measures for the implementation component of the trial

The primary independent variable is study arm (Arm I: tool + basic training vs. Arm II: tool + enhanced training + facilitation). The primary outcome measures are directed by the widely accepted RE-AIM (Reach, Effectiveness, Adoption, Implementation, Maintenance) framework for evaluation of implementation success [49]. Reach will involve rates of insured patient visits and rates of insurance continuity. Effectiveness will refer to rates of patients receiving guideline-concordant cancer screening and other preventive care. Adoption will pertain to patterns, frequency, and timing of insurance support HIT tools usage by clinic staff and patients. Implementation will relate to users’ perceptions (perceived ease of use, usefulness of receiving information about health insurance), and acceptance of the tools (intention to use and satisfaction with the tools). Maintenance will involve all of the above measures over time. Some of these outcomes overlap with those of the effectiveness component of the trial.

### Analytic strategy

#### Quantitative analysis of the effectiveness component of the trial

We will estimate pre- and post-implementation rates of insurance coverage and cancer screenings for clinics in the intervention group and the matched control group. For each patient in the study population, we will determine continuity of coverage and receipt of age- and gender-appropriate recommended cancer screening and preventive care 18 months pre- and 18 months post-intervention. The intervention group will be compared with the matched control group using a difference-in-differences (DID) approach. We will utilize generalized linear/non-linear mixed models, which offer flexible regression modeling to accommodate different sources of correlations (serial and intra-clinic), categorical and continuous covariates, and fixed and time-dependent covariates. This general model allows us to study a wide range of dependent variables, including logistic regression (binary data), beta regression (percent data), Poisson regression (count data), and Gaussian regression (normally distributed data). For example, we will use a random-effect logistic regression model to analyze insurance continuity at pre- and post-intervention periods (dependent variables) as a function of whether a patient belongs to a control or intervention CHC (primary independent variable) and other possible confounders. Serial and intra-clinic correlations will be modeled as random effects.

#### Quantitative analysis of the implementation component of the trial

Similar to the effectiveness aspect of the trial, we will compare the pre- and post-intervention rates of patients’ insurance coverage and receipt of recommended cancer screenings using DID generalized linear/non-linear mixed models comparing Arm I and Arm II. Moreover, post-implementation, we will assess and compare use of the tools across the study arms, including information such as who uses which tools and functions, as well as frequency and timing of use. This will help identify individual- and clinic-level factors associated with more frequent use of the health insurance support HIT tools.

To evaluate tool use, we will assess monthly data (e.g., percent of patient charts with evidence of tool use) in regression models to estimate associations between use and patient panel characteristics. Additionally, we will describe and compare the characteristics of patient encounters with tool use vs. those with no tool use, via random-effect logistic regression models that assess associations between use of the insurance support HIT tools (dependent variables) and socio-demographic characteristics (independent variables) and other possible confounders.

#### Qualitative analysis

Our team will meet regularly to review transcripts and field notes. We will also listen to and discuss key segments of the audio-recorded interviews. This step is crucial to monitoring data quality, refining the observation and interview guides, making sampling decisions, and monitoring theme saturation. This ongoing process will be used to track emerging themes and to create a coding template for more in-depth analysis. We will follow the 5-phase analysis strategy described by Miller and Crabtree (describing, organizing, connecting, corroborating/legitimizing, representing) [50, 51]. To accomplish these steps, we will use an immersion-crystallization approach in which the team reads and discusses the data for each clinic (immersion) to identify key findings (crystallization) [52, 51]. We will do this two times: first, to identify key themes within each case (clinic) and second, to identify cross-case finding. A key step in this process is the connecting phase where we will connect what we are seeing in the qualitative data with the data usage patterns from the EHR, as well as with quantitative data.

## Discussion

Recent policies have created new opportunities for CHC patients to obtain health insurance coverage. However, research indicates that simply making insurance available is not enough to improve rates of continuous coverage; outreach efforts are needed to keep eligible patients insured [53–60]. Multi-strategy approaches have shown early promise in improving enrollment and retention of eligible children in public health insurance programs, but few technological solutions have been tested in primary care settings or with populations of adults eligible for Medicaid coverage [61, 62]. With the passage of the ACA, CHC patients in states that expanded Medicaid now have better access to insurance coverage. Medicaid enrollment increased by 12.9 % in expansion states and by 2.6 % in non-expansion states [63].

The growth of HIT infrastructure in CHCs has created new opportunities for building tools capable of supporting health insurance enrollment and retention. While clinic-based HIT tools (e.g., registries, “pop-up” alerts, automated e-mails to patients) have proven effective at enhancing chronic disease management [64], we know of no previous studies to test the use of similar mechanisms to connect adult patients to Medicaid coverage with the aim of improving insurance coverage continuity and cancer screening rates. Therefore, this study will test the effectiveness of these tools and compare different strategies for supporting their implementation in “real-world” practice settings.

This protocol has several limitations. First, a participant site could withdraw from the study; however, the large number (>300) of OCHIN member CHCs could provide substitute sites. Second, as with any study conducted in “real-world” settings, unobserved changes will undoubtedly occur over time in a non-random fashion within the study environments (e.g., community health insurance outreach efforts). We acknowledge that these unexpected occurrences may make it difficult to isolate the effect of the health insurance support HIT tools and the implementation support strategies in this pragmatic trial. To address this, our approach uses both clinic- and individual-level comparisons. Further, the implementation component of this trial is strengthened by the use of a two-arm cluster-randomized design, and the tool effectiveness component uses a propensity score-matched control group of clinics to adjust for temporal trend. Third, while EHR data sources are not developed for research purposes, we have conducted multiple validation studies and have successfully built research datasets in the past [31–69]. Finally, as with most implementation research, there are questions about sustainability. Conducting this work in partnership with OCHIN positions us to sustain the aspects of the insurance support HIT tools proven effective. OCHIN is committed to maintaining the tools and will also enable rapid dissemination to more CHCs.

Despite these limitations, this study has great potential impact, given the central role played by CHCs in providing health care to vulnerable populations and the expansion of both CHCs and public health insurance coverage for adults as supported by the American Recovery and Reinvestment Act and the ACA [70]. Indeed, as health insurance expansions continue, it becomes increasingly important to know about effective methods for connecting patients to coverage. The proposed study will contribute to this knowledge gap. We believe our findings will have broad relevance to public health and healthcare reform efforts. We will work with our community partners and state policymakers to widely distribute findings and plan for widespread dissemination of the insurance support HIT tools, if proven effective. Further, the insurance support HIT tools have the potential to facilitate access to a wide range of recommended healthcare services beyond cancer screening and prevention.

### Trial status

The recruitment and randomization into the study arms have been completed. We also identified the control group using the propensity-matched score method. The implementation of the health insurance support HIT tools is scheduled for Fall 2015.

## Abbreviations

ACA: Affordable Care Act
CHC: community health center
DID: difference-in-differences
EHR: electronic health record
FOBT: fecal occult blood test
HIT: health information technology
HPV: human papillomavirus
LMS: learning management system
